# Pathogens electrogenicity as a tool for in-situ metabolic activity monitoring and drug assessment in biofilms

**DOI:** 10.1016/j.isci.2021.102068

**Published:** 2021-01-19

**Authors:** Waheed Miran, Divya Naradasu, Akihiro Okamoto

**Affiliations:** 1 International Center for Materials Nanoarchitectonics, National Institute for Materials Science, 1-1 Namiki, Tsukuba, Ibaraki 305-0044, Japan; 2 Graduate School of Chemical Sciences and Engineering, Hokkaido University, North 13 West 8, Kita-ku, Sapporo, Hokkaido 060-8628, Japan

**Keywords:** Multi-drug resistant organisms, microbiofilms, bioelectronics, cell biology

## Abstract

Concerns regarding increased antibiotic resistance arising from the emergent properties of biofilms have spurred interest in the discovery of novel antibiotic agents and techniques to directly estimate metabolic activity in biofilms. Although a number of methods have been developed to quantify biofilm formation, real-time quantitative assessment of metabolic activity in label-free biofilms remains a challenge. Production of electrical current via extracellular electron transport (EET) has recently been found in pathogens and appears to correlate with their metabolic activity. Accordingly, monitoring the production of electrical currents as an indicator of cellular metabolic activity in biofilms represents a new direction for research aiming to assess and screen the effects of antimicrobials on biofilm activity. In this article, we reviewed EET-capable pathogens and the methods to monitor biofilm activity to discuss advantages of using the capability of pathogens to produce electrical currents and effective combination of these methods. Moreover, we discussed EET mechanisms by pathogenic and environmental bacteria and open questions for the physiological roles of EET in pathogen's biofilm. The present limitations and possible future directions of *in situ* biofilm metabolic activity assessment for large-scale screening of antimicrobials are also discussed.

## Graphical abstract

**Figure:**
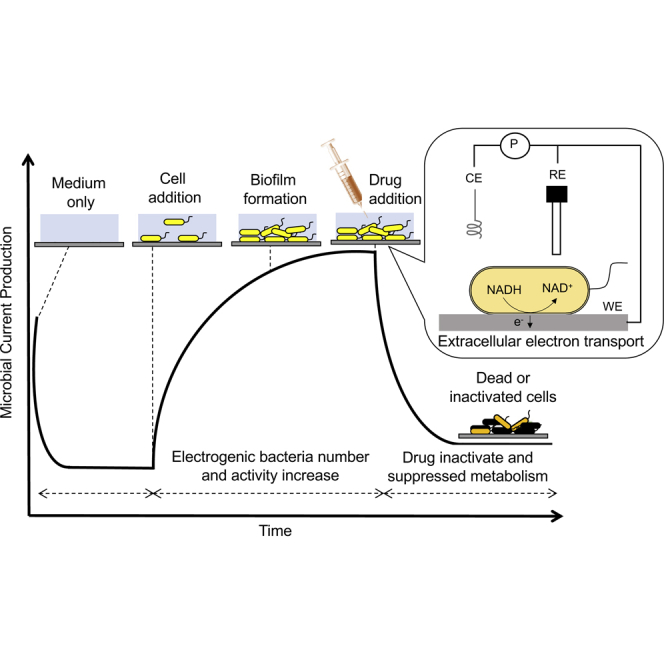

## Introduction

Biofilms formed by microbial communities are considered to be one of the most widely distributed and successful modes of survival on earth (Flemming et al., 2016; Stoodley et al., 2002). Compared with free-living bacteria, those living in biofilms exhibit emergent properties, including social cooperation, resource capture, as well as enhanced survival following exposure to antibiotics and rapid recovery from physical removal (Costerton et al., 1987; Dufour et al., 2010). Although the management of biological energy production and energy cooperation is well studied in environmental bacteria, only little is known regarding this in pathogenic biofilms. Furthermore, misuse of antibiotics has increased the risk of pathogens' infections, which are becoming essentially untreatable due to the acquisition of resistance to currently available antibiotics (Andersson and Hughes, 2011; Brown and Wright, 2016). As the treatment of many infections involving pathogen-containing biofilms is already a formidable problem, and such infections have high mortality rates and create huge economic burdens (Wu et al., 2015), the development of new antibacterial agents and methods to assess the effectiveness of new therapeutic strategies are high in demand.

**Figure 1:**
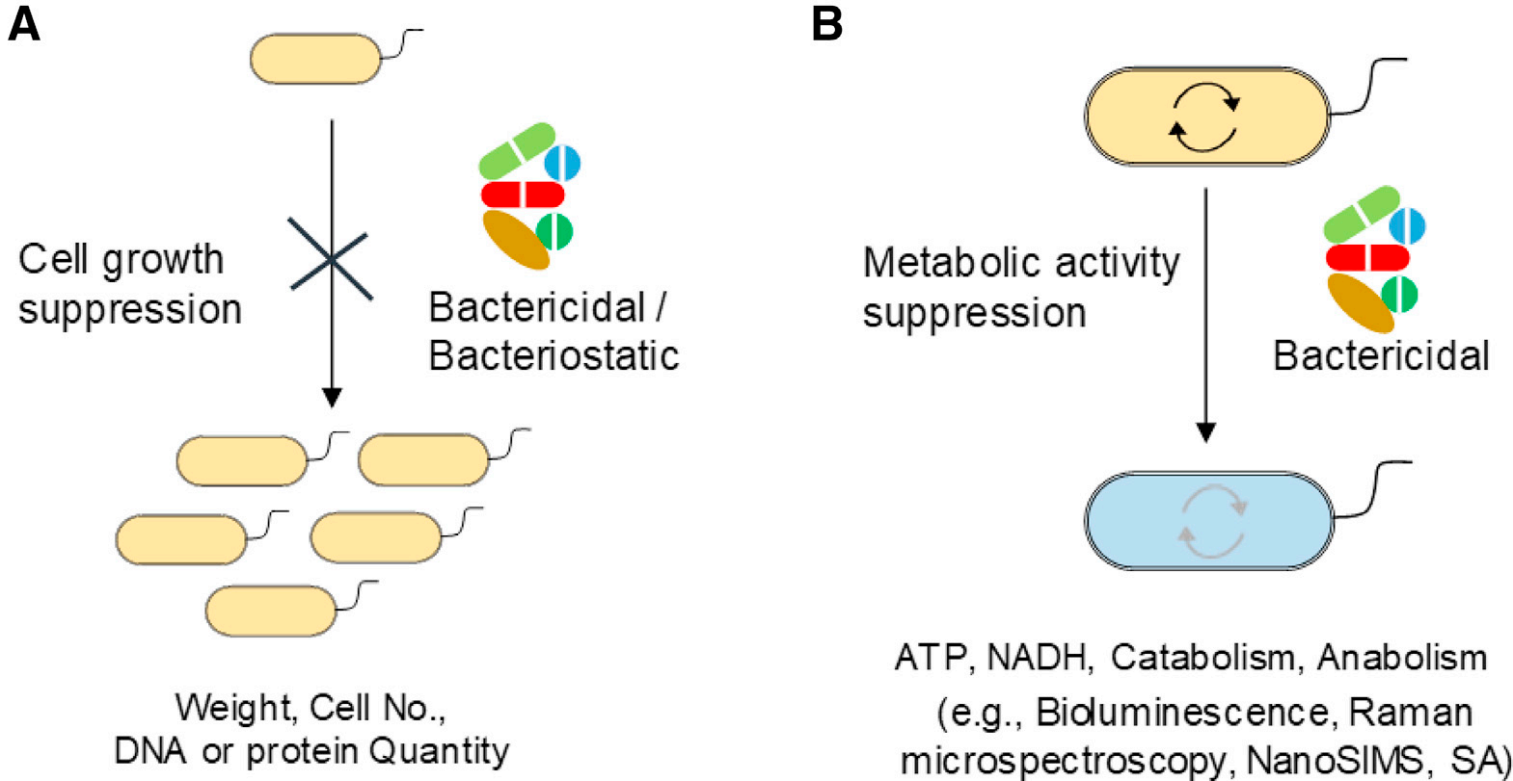
Comparison of growth-based versus non-growth-based techniques (A and B) (A) Growth-based and (B) non-growth-based techniques are employed for antimicrobial drugs assessment. Non-growth techniques have several advantages over growth-based techniques, as they are rapid, suitable for nonculturable microbes, and reliable for non-growing but metabolically active cells. The non-growth-based approaches evaluate specific cellular chemical process such as isotope accumulation, gene upregulation, or metabolic redox reactions.

The development of advanced techniques to assess new antibacterial agents targeting the metabolic activities of pathogens is critical, as conventional growth-based methods require long exposure times, cannot be used for non-culturable microbes, and are unable to discriminate between bactericidal and bacteriostatic effects (Figure 1) (Christensen et al., 1985; Hartree, 1972; Ishiki et al., 2018; Manina et al., 2015; Mosmann, 1983; Richards et al., 2020; Tao et al., 2017; Walker and Keevil, 1994). Technologies exist to assess the metabolic activities of biofilms labeled with isotope or fluorescent substrates, such as single-cell Raman spectromicroscopy and adenosine triphosphate (ATP) bioluminescence (Lee et al., 2017; Stepanenko et al., 2008; Takenaka, 1994; Wang et al., 2020). However, none of these techniques allow real-time monitoring of the metabolic activities of cells in biofilms without labeling.

Recently, some well-known human pathogens were shown to be electrogenic, and correlations between the metabolic activities of the biofilm and electrochemical signals have been demonstrated. For example, *Listeria monocytogenes* (*L. monocytogenes*), a food-borne human gut-associated pathogen, can produce an electric current (Table 1), which is correlated with the extent of biofilm formation (Light et al., 2018). Similarly, *Streptococcus mutans* (*S. mutans*)), an oral pathogen, was also found to be electrogenic, and production of electric current was correlated with single-cell metabolic activity in the biofilm formed on the working electrode (Naradasu et al., 2020a). Notably, Naradasu et al. showed that addition of a metabolic inhibitor immediately decreased current production. These observations led to the development of a real-time assay for drug assessment by directly measuring the current production associated with cellular metabolism in biofilms. In these assays, microbial current production is measurable using disposable, low-cost electrode systems and single-potential amperometry (SA) techniques, and changes in current reflect the impact of drugs on metabolic activity. This novel technique for drug assessment does not require cellular growth and thus, can be used to evaluate effects on non-culturable bacteria as well (Figure 1). Although many recent studies have reported electrogenic activity in human pathogens during biofilm formation in pure cultures of pathogens and in animal or human microbiomes (Table 1), the correlation between biofilm metabolic activity and current production was not the main focus of these studies. Herein, we review EET-capable pathogens and the methods to monitor the biofilm activity to discuss the advantages of SA-based drug assessment and propose techniques that can be used in combination with SA to maximize information about pathogenic biofilm activity. We further focus on the range of microbial diversity that can potentially be assessed with SA-based techniques, the prevalence of extracellular electron transport (EET) among pathogenic bacteria, and many open questions regarding EET in pathogens. Finally, we will discuss the other potential biosensor applications which can be built by using pathogen's current producing capability.

**Table 1:** EET capable pathogens and critical factors for SA measurements

Organism	Niche	Gram staining	Electron donor	Electron acceptor	Applied potential (mV versus SHE)	Current generation (μA cm^−2^)	Biofilm confirmation	Observed redox potential (surface/mediator) (mV versus SHE)	Biofilm structure	Assay for metabolism correlation	Reference
*Listeria monocytogenes*	Human gut	+ve	glucose	Graphite felt	+600	23	CFU	+77^a^	–	Gene deletion	(Light et al., 2018)
*Enterococcus faecalis*	Human gut	+ve	glucose	Graphite rod	+588	23 to 43	CLSM	−25^b^ (menadione)	Multilayer	Gene deletion	(Pankratova et al., 2018) Keogh et al. (2018)
*Pseudomonas aeruginosa*	Human lungs	−ve	glucose	Graphite rod	–	2	–	−180^b^ (pyocyanin)	–	Gene deletion	(Rabaey et al., 2005)
*Klebsiella pneumonia*	Human gut	−ve	glucose	ITO electrode	+400	0.13	SEM	−50^a^	Monolayer	–	(Naradasu et al., 2018)
*Enterococcus avium*	Human gut	+ve	glucose	ITO electrode	+400	0.12	SEM	−350^a^	Monolayer	–	(Naradasu et al., 2018)
*Streptococcus mutans*	Human oral	+ve	glucose	ITO electrode	+400	0.050	SEM	−50^a^ & +250^a^	Multilayer	Drugs/NanoSIMS	(Naradasu et al., 2020a)
*Aggregatibacter actinomycetemcomitans*	Human oral	−ve	lactate	ITO electrode	+400	0.065	SEM	−60^a^	Monolayer	Drugs	(Naradasu et al., 2020b)
*Porphyromonas gingivalis*	Human oral	−ve	glucose	ITO electrode	+400	0.016	SEM	+150^a^	Monolayer	Drugs	(Naradasu et al., 2020a, 2020b)
*Capnocytophaga ochracea*	Human oral	−ve	glucose	ITO electrode	+400	0.060	SEM	−40^a^	Multilayer	Cell density	(Zhang et al., 2020b)
*Corynebacterium matruchotii*	Human oral	+ve	glucose	ITO electrode	+400	0.050	SEM	−125^a^ & +25^a^	Multilayer	Drugs/NanoSIMS	(Naradasu et al., 2020c)
*Clostridium cochlearium*	Mouse gut	+ve	glucose	Graphite rod	+700	530	–	+420^b^ (unknown)	–	–	(Schwab et al., 2019)

a Redox peak was most likely from surface proteins.

b No surface redox peak was observed. Redox peak was from cell-secreted mediators.

## Single-potential-amperometry-based quantitative assessment of antibiotic reagents for pathogen biofilms

Electrochemical assessment of pathogenic biofilms has been carried out with electrochemical impedance spectroscopy (EIS) and cyclic voltammetry (CV) to sense the presence of bacteria on electrodes, as described in the later sections. In contrast, SA had scarcely been used to analyze the impact of antibiotics prior to the discovery of electrogenic pathogens. As shown in Table 1, various pure bacterial cultures were experimentally shown to have current-producing capability on electrodes. Several well-investigated pathogens have emerged as electrogenic, and the extent of current production (*I*_*c*_) is at the micro to nano ampere level, which is still in orders of magnitude higher than the detection limit of electrochemistry. These identified current producing bacteria are mainly gut or oral pathogens that may cause serious illnesses. Also, pathogens such as *Klebsiella pneumoniae* and *Pseudomonas aeruginosa* are known to be drug resistant and are in focus as targets for drug discovery owing to their biofilm infections (Doorduijn et al., 2016; Moradali et al., 2017).

The capability of current production is often explained by the electron transport process from the cell-surface redox protein, referred to as the EET mechanism. However, production of current can also occur without an EET mechanism, and only a few strains have been shown or suggested to have EET capability. Nevertheless, even with EET or not, drug assessment is possible and reliable with a good correlation between microbial current and cellular metabolic activity, as current production capability appears to support the growth of fermentative pathogens on the electrode surface. Although not all the current producing pathogens (Table 1) reported so far have enough evidence, the points mentioned below are important to confirm the applicability of SA-based biofilm drug assessment to current-producing pathogens of interest.

**Figure 2:**
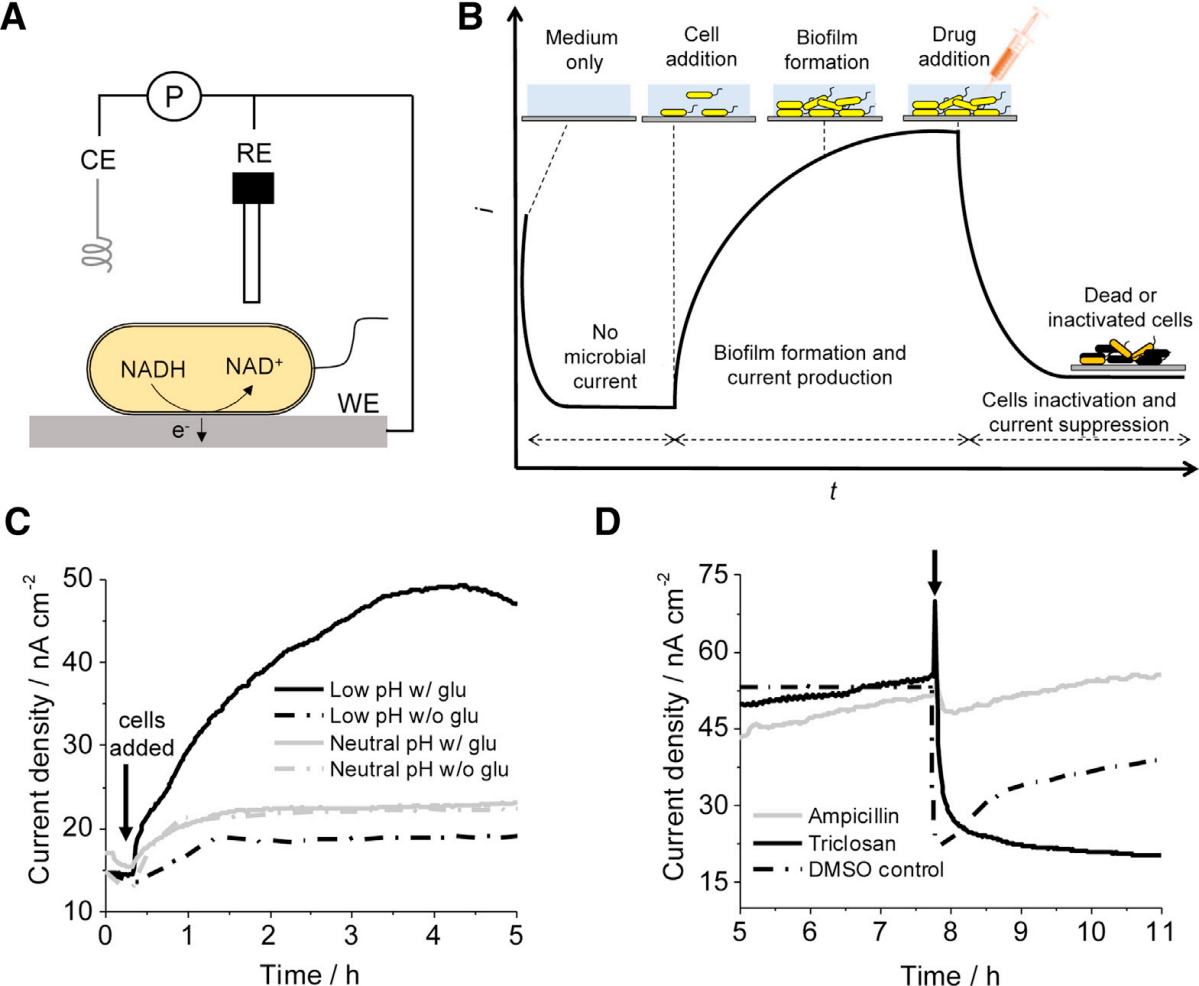
Single potential amperometry (SA) for quantitative assessment of antimicrobial reagents for pathogen biofilms (A) Schematic representation of a three-electrode electrochemical system used for SA measurements. (B) Schematic representation of the time course of the microbial current production and the effect of the addition of drugs at a fixed electrode potential. (C) Current production versus time measurements with ITO electrodes poised at +400 mV (vs SHE) in the presence and absence of glucose with *S. mutans* pregrown at low pH (4.6 ± 0.2) and neutral pH (7 ± 0.2). (D) Effect of antibiotics on current generation. Ampicillin, an inhibitor of cell wall biosynthesis (gray line), and triclosan, a metabolism inhibitor (black line), were added at the points indicated by the arrow to test their impacts on *S. mutans* electrochemical activity. DMSO control (black dash dot line): DMSO (same volume as in triclosan treatment, black line) was added to test the impact of solvent on current generation (Naradasu et al., 2020a).

SA is a simple technique to count the number of electrons moving between the microbial biofilms and the electrode surface at a fixed electrode potential with the output of the time course of the microbial current production (Figure 2). This technique requires a three-electrode system, where the potential of the working electrode (WE) is poised to a certain value against the reference electrode and the counter electrode compensates for the charge transfer at the WE (Figures 2A and 2B). It is vital to select the proper ranges for the potential and current to achieve good accuracy, as too positive or negative poised potentials may result in oxidization or reduction of the reactor medium components or the electrode itself. The potential range without background reactions is referred to as the potential window of the electrode. If the WE electrode gets corroded or redox-active compounds are included in the electrolyte, a high background current might hamper the detection of biotic signals even within the potential window. Therefore, it is important that the stability of the WE and electrolyte is confirmed prior to the experiment with the biofilm. As shown in Table 1, SA measurements with different pathogens are mainly conducted at +400 – +600 mV versus standard hydrogen electrode (SHE), which is mostly about 200–300 mV more positive compared with redox reagents found in biological electron transport chains. The electrodes are composed of graphite or indium tin oxide (ITO), and the electrolyte is a minimum medium or rich medium such as brain–heart infusion broth.

Once SA measurement starts with the electrolyte in the absence of microbes, the background current is observed. Usually, it becomes stable after a few minutes or hours, depending on the component of the medium (Figures 2B and 2C). Microbes are then introduced into the electrochemical reactors, and the presence of electrogenic bacteria can increase the current associated with the consumption of electron donor substrates such as glucose (Table 1). Direct evidence of electrogenic capability is therefore the obvious current increase in the presence of the electron donor. It is also important to check the inertness of the electron acceptor (electrode) to avoid any error in the current production values. The control experiments were conducted for all the strains in Table 1 to confirm the association of current generation with electron donor oxidation. In case of fermentative EET pathogens, the minimum medium with glucose gave only 50 nA cm^−2^ (*S. mutans*) (Table 1). In contrast, *L. monocytogenes* and *Clostridium cochlearium* showed much higher current in different conditions with rich medium or highly positive electrode potential. Further investigation is required to determine whether this difference was due to microbes, amount of nutrients, metabolism, or electron transport mechanism.

**Figure 3:**
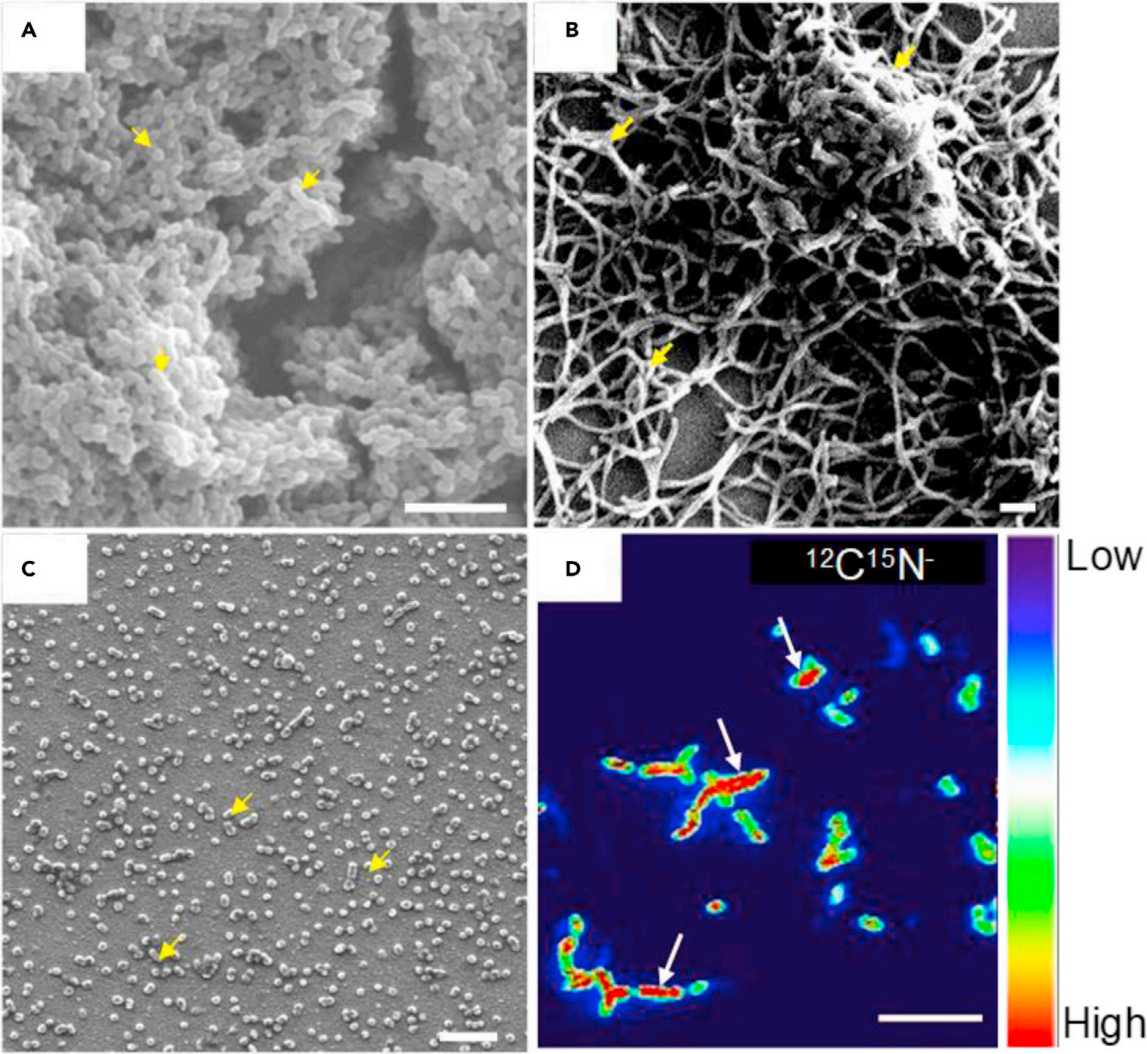
Microscopic observations of electrogenic biofilms and single cell metabolic activity (A–C) Scanning electron micrographs showing multilayered biofilm formation by (A) *S. mutans* and (B) *C. matruchotii,* and monolayer by (C) *P. gingivalis* on the ITO electrode after multiple washings. Scale bar, 5 μm (Naradasu et al., 2020a, 2020b, 2020c). (D) NanoSIMS image of cells attached to the ITO electrode showing the ^12^C^15^N^—^ ion pixel intensity for *S. mutans*. Scale bar, 5 μm. Color gradient bar indicates ion pixel intensity. Arrows indicate the representative cells (Naradasu et al., 2020a).

It is also important to mention that the current production often increases with time for at least a few hours, as it is supported by the growth of electrogenic bacteria. After a certain time period, bacteria often form monolayer or multilayer biofilms (Table 1). For example, *S. mutans* and *Corynebacterium matruchotii* (*C*. *matruchotii*) produced multilayered biofilms after 8 h of incubation, whereas *Porphyromonas gingivalis* (*P. gingivalis*) and *Aggregatibacter actinomycetemcomitans* (*A. actinomycetemcomitans*) formed only a monolayer biofilm after 24 h of incubation. Scanning electron microscopy (SEM) can be used to confirm the biofilm formation (Figures 3A–3C), as SEM sample underwent several washings and it helped to remove planktonic or weakly attached cells from the electrode.

The impact of antibiotics on the metabolic activity of the biofilm should be assessed once the current gets stabilized in the presence of the biofilm on the electrode (Figure 2B). If the substrate is effective for suppressing the metabolic activity, current production should decrease in a few minutes, as shown in Figures 2B and 2D. Because the mechanism used by the pathogen for current production is not completely clear, it is important to confirm whether the suppression of metabolic activity caused the decrease in the current. The most facile method would be the addition of inhibitors for specific metabolic processes during SA. For example, the addition of ampicillin inhibits cell wall formation and ultimately affects cell growth, and triclosan, a glycolysis inhibitor, at lethal concentrations results in a significant decrease in the growth of *S. mutans*, and *C. matruchotii* (Naradasu et al., 2020a, 2020c) (Table 1). It is important to mention that only lethal concentrations have been tested for antibiotic dosage in pathogen's current-producing reactors, and more detailed studies will help in establishing the dose-dependent behavior of current production.

Further evidence of correlation between current production and metabolic activity was obtained from gene-deletion mutations and isotope-based single-cell analysis carried out on *L. monocytogenes* and *S. mutans*, respectively (Table 1). In *L. monocytogenes,* the mutant strain lacked current production capacity and showed significant deficiency in biofilm formation in mouse gut. Nano-scale secondary ion mass spectrometry (NanoSIMS) was used for localization and viability of the cells on the electrode and to quantify the assimilation of isotopically labeled nutrient (Figures 3D), ^15^NH^4+^, which couples with ATP and NADH production via metabolism. High ^15^N assimilation in *S. mutans* cells with high *I*_*c*_ confirmed the correlation between the *I*_*c*_ and metabolic activity of single cells in the biofilm.

Because decreased antibiotic susceptibility is also associated with reduced antibiotic penetration, new drugs are being designed to enhance biofilm penetration and reach its bottom. In this aspect, our proposed model could also be effective because electroactive biofilms may favor growth at the biofilm–electrode interface, and most active cells may be found at the electrode surface (Chadwick et al., 2019); hence, the effectiveness of drug penetration can be combined with electroactivity. Based on all these points, the spectrum of pathogens that can apply the SA method to confirm their current production capability can be expanded by our protocol.

Other than human pathogens, corrosion-causing and associated bacteria such as *Desulfovibrio vulgaris* and *Thioclava electrotropha* *ElOx9* have also shown the EET capability, and current produced by these bacteria has been found to be coupled with cellular metabolism (Deng et al., 2020; Karbelkar et al., 2019). Collectively, these studies suggest that the assessment of new drugs/metabolic inhibitors is possible by employing the current production capability of harmful bacteria such as human pathogens and iron corrosion bacteria.

## Potential techniques in combination with SA to assess antibiofilm drug

Although the SA technique explained above is facile and provides a direct assay for the metabolic activity of label-free biofilms attached to the electrode surface, the decrease in microbial current may not be fully explained by metabolic activity, but possibly by the reduction of cell number on the electrode. To distinguish these two situations, various techniques can be combined with SA to obtain a deeper insight. For example, SEM and NanoSIMS were applied to the biofilm on the electrode to confirm the biofilm morphology and the single-cell activity, respectively, in Figure 3 as discussed earlier. Here, we showcase some options in Table 2 and highlight some techniques.

**Table 2:** Evaluating process, detection limit, advantages, and disadvantages of SA and combinable techniques with SA for biofilm analysis

Techniques	Evaluating process/detection limit	Advantage	Disadvantage	Reference
Electrochemical impedance spectroscopy	Electrode resistance and capacitance change by electrode cell coverage	Direct correlation with biofilm formation; non-destructive	Not suitable for electrodes with large capacitance; limited throughput	(Fricke, 1933; Yoho et al., 2015)
Colony counting	Cell growth on solid medium	Direct correlation with live cell number; low cost and high throughput	Biofilm homogenization or flow system required; only applicable to culturable bacteria on solid media	(Mansberg, 1957)
Total protein quantification	Biofilm formation/4 μg mL^−1^	High throughput; direct quantification of biomass in biofilm	Dead cell detection	(Hartree, 1972; Richards et al., 2020)
Flow cytometry	Cellular detachment from biofilm/0.5 μm	Direct cell counting; labeling applicable	Biofilm homogenization or flow system required	(Bogachev et al., 2018; Kerstens et al., 2015; Steen and Boye, 1980)
Biofilm dry mass	Biofilm formation	Directly applicable to biofilm	Large biofilm required; limited sample throughput;	(Characklis et al., 1982)
Electrochemical tetrazolium salt assay	Intracellular redox reaction activity/>2.8 × 10^1^ CFU mL^−1^	Directly applicable to biofilm	Only electrode adsorbed molecules can be detected	(Ishiki et al., 2018; Mosmann, 1983)
Crystal violet assay	Biofilm formation	High throughput; direct quantification of cell number in biofilm	Only applicable to thick biofilm; dead cell detection; large variations in result	(Christensen et al., 1985)
Scanning electron microscopy	Biofilm formation and cell morphology/>1 nm	High-resolution image of cells and nano structures in biofilm	Time consuming and low throughput	(Danilatos, 1988; Golding et al., 2016)
Fluorescent microscopy/confocal fluorescent microscopy	Fluorescent intensity in cell or biofilm/>1 μm	Fluorescent proteins can specifically tag various and multiple cellular processes	Gene engineering required; complex factors to change fluorescent intensity	(Lawrence et al., 1991; Palmer et al., 2006; Zhang et al., 2020a, 2020b)
Nuclear magnetic resonance	Biofilm formation and metabolism in biofilm	Live, non-invasive monitoring of chemicals in biofilm	Large biofilm required; low throughput	(Lewandowski et al., 1992; McLean et al., 2008)
Quartz crystal microbalance	Cellular attachment to sensor surface/1 pg	Non-destructive; high sensitivity to molecular level	Limited throughput; indirect cell counting	(Dixon, 2008; Nivens et al., 1993; Olsson et al., 2015)
Light microscopy	Single cell growth/>1 μm	Real-time quantification of cell growth and motility	Not suitable for multilayer biofilm; limited throughput	(Walker and Keevil, 1994)
Adenosine triphosphate bioluminescence	ATP content of the biofilm/10^2^ CFU mL^−1^	Nondestructive, rapid; real-time assay; cost-effective	Not suitable for low activity cells; high interference	(Lee et al., 2017; Takenaka, 1994)
Raman microspectroscopy	Isotope accumulation via cell metabolism/1 wt%, >1 μm	Single cells activity achievable; real time monitoring	Not suitable for multilayer biofilm; limited throughput	(Chisanga et al., 2018; Ivleva et al., 2017; Marcotte et al., 2004)
Nano scale secondary ion mass spectrometry	Isotope accumulation via cell metabolism/>0.1 wt%, >50 nm	Single cells activity achievable; highly sensitive and quantitative	Not suitable for multilayer biofilm; expensive and time consuming	(Behrens et al., 2008; Musat et al., 2016; Renslow et al., 2016)
Cyclic voltammetry	Cellular secretion of redox substrates or cell outer membrane redox active proteins/10 nM	Non-destructive; no labeling required	Indirect cell quantification; limited throughput	(Bai et al., 2010; Harnisch and Freguia, 2012; Marsili et al., 2008)
Electrochemical Quartz crystal microbalance	Cellular attachment to electrode surface/1 pg	Non-destructive; high sensitivity to molecular interaction	Limited throughput; indirect cell counting	(Babauta et al., 2014; Brown-Malker et al., 2010)
3D magnetic resonance imaging	Biofilm formation and chemical reaction/28 μm	Non-destructive; various chemicals traceable	Limited throughput; large biofilm required	(Caizán-Juanarena et al., 2019; Renslow et al., 2013)
Single potential amperometry	Extracellular electron transport associated with metabolic activity/>1 nA cm^−2^	Real-time quantification of cellular metabolic activity; non-destructive	Applicable to current producing bacteria only; limited throughput	(Naradasu et al., 2020a, 2020c)

### Electrochemical impedance spectroscopy

Other electrochemical techniques can be applied often at a software interface for the same potentiostat along with SA. EIS and CV are excellent candidates for this purpose. Although originally applied to the investigation of solid–solid interfaces, EIS is a common and promising method for the assessment of pathogenic species and biointerfaces without the requirement of any additional reagents and allows label-free sensing (Fricke, 1933; Yoho et al., 2015). In most EIS methods, samples are subjected to a small sinusoidal potential perturbation. The frequency of this perturbation is shifted in the range between a few mHz and 10^5^ Hz. The subsequent sinusoidal current is evaluated by fast Fourier transform techniques to estimate the impedance (Z) of the interface in the frequency domain to measure diffusion at surfaces covered by protein monolayers (Chi et al., 2000), charge transfer time constants (Repo et al., 2000), electron transfer mechanisms (Bai et al., 2006), and quantification of bacteria (Radhakrishnan and Poltronieri, 2017).

Biofilm growth on conventional electrode systems (such as Au) is known to produce poor signals owing to the small size of microbial cells. However, it has recently been demonstrated that modified electrodes through facile electrochemical activation provide a large surface area for the growth of microbial biofilms and can therefore produce a strong impedance signal in response to a change in the biomass. Song et al. cultured and studied three oral bacteria, *Streptococcus mutans, Actinomyces viscosus*, and *Lactobacillus fermentum*, on the surface of reduced graphene oxide-carbon electrode (rGO-CE) and found that the impedance response signal by pathogen biofilms growing on rGO-CE was many fold stronger than that of the Au electrode (Song et al., 2020).

EIS allows the cost- and power-effective multiplexing and miniaturization of the assessment system, making them suitable for point-of-care diagnostics and the detection of biological agents (Suni, 2008). However, although EIS responds differently for turnover and non-turnover conditions, it is difficult to develop a quantitative correlation between metabolic activity and biofilm formation (Babauta and Beyenal, 2014). This is because EIS is influenced by multiple factors such as the microbial production of redox compounds and metabolites and the attachment of cells, microbial nanowires, protein, and nucleic acid to the electrode surface (Ward et al., 2014). Therefore, although impedance curves obtained for pathogens in wild-type and mutant strains with less biofilm-forming ability or addition of drugs to suppress biofilm formation exhibited distinct phases (van Duuren et al., 2017), a clear correlation is lacking in EIS studies. In contrast, because microbial current production in SA corresponds to metabolic activity, metabolic suppression immediately results in the current decrease. Nevertheless, given that EIS is measurable in the same electrode system for cellular localization, a combination of SA measurement and EIS is a strong methodology to assess the impact of antimicrobial agents and for biofilm characterization on electrodes.

### Cyclic voltammetry

CV-based monitoring of biofilms detects the interfacial electron transport with electrode surfaces but not the catalytic current from metabolic reactions in current-producing bacteria. In CV, the electrode potential is scanned in the forward and reverse directions between two potentials, typically within the potential window of the electrode material, and current values are plotted as a function of electrode potential. A pair of current peaks in forward and reverse scans specifically observed in the presence of microbes, but not in the sterile electrolyte, suggests that some redox species are produced or possessed by microbes that exchange electrons with the electrodes.

CV is shown to be a more appropriate technique than EIS when voltammetric waves do not merge and migrate out of the potential window (Marsili et al., 2008). Vieira et al. developed an electrochemical detector on platinum electrodes based on CV for monitoring the formation of *Pseudomonas fluorescens* (*P. fluorescens*) biofilms *in situ* in batch systems. A three-electrode system was used because the response to the application of the potential profile to the working electrode was highly sensitive to the amount of biofilm deposited on the surface with repetitive cyclic voltammetry (Vieira et al., 2003). The response difference between the uncolonized electrode and the *P. fluorescens* biofilms of different ages grown on its surface showed that cyclic voltammetry applied to platinum electrodes can be used to detect young biofilms.

Given the application of potential could disrupt biofilms either by producing hydrogen (Gião et al., 2005) or by inducing unfolding/oxidation of adsorbed proteins at oxidizing potentials (Perez-Roa et al., 2006), application of CV coupled with SA to living bacteria will require limiting the potential range to prevent harmful oxidizing or reducing conditions as well as selection of informative scan rates (Bai et al., 2010; Harnisch and Freguia, 2012).

### Scanning electron microscopy

SEM is a very useful tool not only for detailed observation of the substratum morphology but also to confirm bacterial adhesion and biofilm formation on different surfaces subjected to pretreatment of samples that involve multiple washing and drying steps. SEM possesses the level of magnification and resolution, which is required to observe the overall shape of bacteria forming the biofilm as well as to enable their spatial organization (Danilatos, 1988; Golding et al., 2016; Hannig et al., 2010; Norton et al., 1998). This type of spatial analysis offered by SEM make it a method of interest for the assessment of biofilm growth on different surfaces, contrary to traditional methods that offer bulk quantifications only. SEM microscopy has already been used to examine and characterize the early age of biofilms on various devices, and it is useful in the development of antibiofilm materials for biomedical applications (Gomes and Mergulhão, 2017; Grenho et al., 2014; Steffensen et al., 2015). Although SEM is not compatible with the use of fluorescent dyes such as Syto9 and propidium iodide, frequently employed for distinguishing between live and dead cells, it still assists in comprehensive observation of single cells in the biofilm and their morphology (Serra et al., 2013). Therefore, the use of SEM (coupled with SA) for high-resolution imaging of colonized surfaces can provide valuable information about the pathogen biofilms when assessed for antibiotic treatment.

### Nano-scale secondary ion mass spectroscopy

NanoSIMS allows single-cell analysis for metabolic activity with high sensitivity and spatial resolution (∼50 nm), which enables the quantification of single-cell activity without disturbing their spatial relationships (Behrens et al., 2008; McGlynn et al., 2015; Musat et al., 2016). In this method, incubation of microbes is carried out with isotopically labeled substrate(s), and samples are fixed on a conductive surface, which is then bombarded with a primary ion beam of Cs^+^ and O^−^ (or rarely O_2_^−^) for negative and positive secondary ion analysis, respectively, in a high vacuum chamber. As a result, fractions of atoms and atomic clusters are obtained from the sample surface, a part of which are ionized and detected as secondary ions (Renslow et al., 2016). Although catabolic processes are not directly monitored in NanoSIMS, the extent of catabolism associated with cellular isotopic enrichment is quantified as an index of metabolic activity. Particularly for biofilm on the electrode, NanoSIMS specifically analyzes cells that strongly attach to the surface of the electrode as planktonic, and loosely attached cells are removed in the sample preparation process. Moreover, this method for single-cell analysis of metabolic pathways can be applied to any EET-capable bacteria, as it does not require gene engineering (Saito et al., 2017, 2020).

### Confocal laser scanning microscopy

Confocal laser scanning microscopy (CLSM) is a specialized form of microscopy that is used to produce high-resolution and sharp images of cellular and polymeric biofilm components in three dimensions (Bjarnsholt et al., 2013; Nwaneshiudu et al., 2012). 3D imaging is achieved because the confocal optics can be focused on a very small volume in the biofilm. The scanning of the focused area across the sample assists in producing high-resolution 2D “slices” at different heights, which are then assembled to produce a final 3D image. Because CLSM usually does not involve the washing and drying steps, biofilm components may interrupt imaging bacterial cells. To this end, flow-system-based CLSM analysis may be more useful where biofilm-forming adherent cells can be visualized, but special care should be taken when dealing with anaerobic pathogens to avoid oxygen ingress in the flow system, and hence experienced and highly trained personnel should be employed to ensure accurate measurement and analysis (Lawrence et al., 1991; Palmer et al., 2006; Zhang et al., 2020a).

It is important to mention the significance of introducing fluorescence to biofilm samples for microscopic analysis. In addition to innate biomolecules such as NADH and NAD(P)H or chlorophyll, which have fluorescent characteristics and can be used in fluorescence microscopy, fluorescent dyes and proteins are largely employed to analyze samples (Niedenthal et al., 1996). Fluorescent dyes are usually fluorescent molecules, known as fluorophores or biomolecules linked to fluorophores, which absorb and emit light when incorporated into biological structures such as biofilms. The emitted light is detected for image generation to analyze biofilm features such as spatial cellular viability, morphology, and function throughout the growth/treatment period in addition to cell counting and tracking real-time biofilm accumulation. Although introducing fluorescence increases costs and sample preparation time, the additional information obtained is very useful for a greater understanding of cellular growth and life within the biofilm (Jakobs et al., 2000). Depending on the requirement, some common classes of fluorescent dyes and proteins that are used for the analysis of biofilms using microscopic techniques include DAPI (4,6-diamidino-2-phenylindole dilactate), FM 4–64, SYTO9, and propidium iodide (Shaner et al., 2005). The disadvantage of many fluorescent dyes and proteins is their potential interference with cellular processes often resulting in toxicity or changes in the cell, which may limit the types of characterizations possible and sometimes make interpretations very complex.

### Cell counting

Although it is not as cost-effective as an electrochemical method, flow-based methods are also good options to combine with SA to study the detailed mechanism underlying the decrease in the current. Flow-based methods are the automated methods for the counting of cells in which cells in liquid culture usually flow through a narrow aperture and are measured as they pass. Among flow-based methods, colony counting (Mansberg, 1957) and flow cytometry are the common methods, both of which require the homogenization of biofilms in the liquid medium (Bogachev et al., 2018; Kerstens et al., 2015; Steen and Boye, 1980). In contrast to colony counting, flow cytometry provides more detailed information about the cells. Cell dimensions, surface properties, metabolic activity, and the state of the cells can also be gathered using cell staining or endogenous fluorescent tags (Müller and Nebe-von-Caron, 2010).

### Quartz crystal microbalance

Quartz crystal microbalance (QCM) is a reliable technique for bacterial biofilm studies because of its flexibility in investigating molecular recognition and surface phenomena, which allows the nondestructive measurement of biofilm accumulation as a function of time (Ripa et al., 2020). Combination with electrochemistry has also been reported for biofilm assay (Babauta et al., 2014; Brown-Malker et al., 2010). The major advantage of this technique is that it allows the monitoring of mass accumulation to ng/cm^2^ accuracy in real time without sample destruction. A major disadvantage of this method is the high cost of specialized equipment, software, and consumables. Another disadvantage is that the resonant frequency used in this system is highly sensitive to changes in temperature and pressure, requiring a constant monitoring and accounting of these variables during data collection.

### Raman microspectroscopy

Raman microspectroscopy achieves comprehensive intrinsic molecular profile in a single cell without destruction, based on the vibrational frequencies of characteristic chemical bonds. Single-cell Raman spectra have been employed to monitor bacterial phenotypic changes, mostly of the macromolecule contents inside the cells, during antimicrobial treatment at either the population or the single-cell level (Schröder et al., 2015). Raman method by using isotopic accumulation such as D_2_O has been developed as a growth-independent approach for measurement of cellular metabolic activity in response to drug treatment at the single-cell level (Tao et al., 2017). In addition, other studies have employed Raman spectroscopy for chemical analysis of biofilms, differentiation of planktonic and biofilm cells, discrimination of diverse species of bacteria in biofilms, and monitoring metabolic characteristics under different physiologic state (Chisanga et al., 2018; Ivleva et al., 2017; Marcotte et al., 2004). Although Raman spectroscopy allows rapid, *in situ*, non-invasive acquisition of chemical and structural information through the generation of fingerprint spectra, it can be effectively employed to support SA-based biofilm monitoring technique.

### Magnetic resonance imaging

Magnetic resonance imaging (MRI), an approach well known from medical diagnostics, is also considered to be suitable for visualization and characterization of biofilms. By detection of the magnetic moments (spins) of hydrogen nuclei in a magnetic field after excitation, NMR visualizes the whole biofilm (Lewandowski et al., 1992; McLean et al., 2008). In the past, MRI techniques for the non-invasive study of live biofilms have been restricted to the measurement of water properties, but it has been extended to the biofilm phenotypic expression and metabolism at different biofilm depth (Majors et al., 2005). Furthermore, three-dimension MRI revealed diffusion coefficients and metabolic activity within the biofilm growing on flat electrodes (Renslow et al., 2013). Therefore, MRI techniques can also be coupled with SA assay to enrich information about electroactive biofilm.

## Electron transfer mechanism less uncovered in electrogenic pathogens than environmental bacteria

Historically, EET is defined as a way of microbial respiration by which they (environmental bacteria such as *Shewanella* spp. and *Geobacter* spp.) can transfer electrons from the cell interior, across the periplasm, and then through the cell-surface to extracellular electron acceptors such as iron (III) or manganese (IV) oxides (Lovley and Phillips, 1988; Myers and Nealson, 1988). Such environmental EET capable bacteria are considered as “good bacteria” with reference to their significance in driving biogeochemical cycling processes of elements in water, soil, sediment, and subsurface environments (Fredrickson and Zachara, 2008; Lovley, 2011; Richardson et al., 2012). These bacteria are critical for biotechnological applications such as degradation of wastewater and solid waste, bioelectricity generation, and production of bulk and fine chemicals as well as biofuels (Logan, 2009; Logan et al., 2019; Nevin et al., 2010; Rozendal et al., 2009) after replacing mineral oxides with electrodes. The key mechanisms that have been proposed for EET in environmental bacteria are via cell-surface *c*-type cytochrome, redox shuttle molecules, or cell extensions termed as pili (or nanowires) (Shi et al., 2016). The filament structure shown to have polymerized chains of heme closely stacked along the micrometer length of the filament established the molecular basis for electronic conductivity in these nanowires (Wang et al., 2019a). For some of the key enzymes involved in EET, their protein structures have been solved, revealing the potential involvement of protons in electron transport kinetics (Edwards et al., 2020). Sulfate-reducing bacteria, *Desulfovibrio ferrophilus* IS5 strain, and *Desulfovibrio vulgaris* involved in iron corrosion have evolved electron transport mechanisms distinct from those in iron-reducing bacteria (Deng et al., 2018, 2020; Karbelkar et al., 2019).

**Figure 4:**
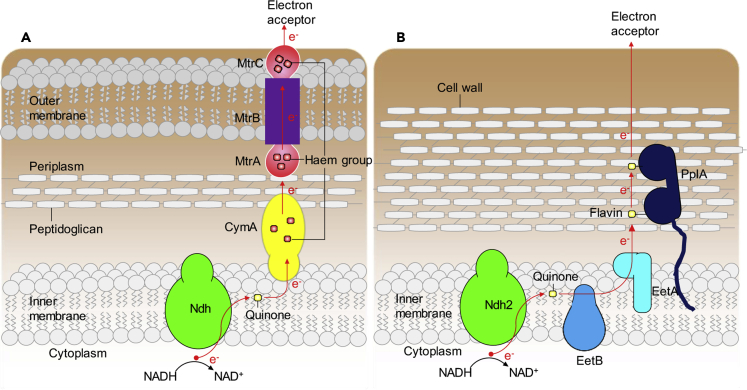
Comparison of identified electron-transfer pathways in environmental and pathogenic bacteria (A) In environmental bacterium *S.oneidensis* MR1, energy is gained by transfer of electron (e^−^) from the NADH to final electron acceptor. In this EET process, the electron-transfer path (red arrow) occurs across two lipid membranes and across the periplasm region, which contains cell-wall material that includes the sugar peptidoglycan. The extracellular electron transfer from the protein Ndh includes a quinone molecule, heme groups associated with the proteins CymA, MtrA, and MtrC, and transfer through the protein MtrB of outer membrane. (B) A distinct EET mechanism is identified in gram-positive pathogen *L. monocytogenes*, which has only a single membrane. The identified components of this EET system includes the proteins Ndh2, EetB, EetA, and PplA associated with two flavin molecules. This newly identified EET process might occur in diverse bacteria, including those in the human gut (Cahoon and Freitag, 2018; Light et al., 2018; Nealson and Rowe, 2016).

In contrast, the EET mechanism in pathogens is far less understood. So far, the important genes have only been well identified in pathogen *L. monocytogenes*, where instead of *c*-type cytochromes, a protein-bound eight-gene locus (including ndh2) coupled with flavins shuttle electrons to the extracellular electron acceptor (Light et al., 2018). The molecular basis of this form of EET is novel with identified proteins Ndh2, EetB, EetA, and PplA as the crucial components of the process. The initial electron-transfer steps of EET in *L. monocytogenes, i.e.*, electron transport steps from the cytoplasm to a quinone pool in the lipid membrane, bear a resemblance to those in mineral-respiring EET model strains. However, the electron transport steps become more distinct beyond this point, as *L. monocytogenes* is a gram-positive bacterium with a single lipid membrane and a thick cell wall, whereas model environmental strain such as *Shewanella* *oneidensis* is a gram-negative bacteria with two lipid membranes separated by the periplasmic region where multiple heme molecules bound to three types of protein provide paths for the electrons to cross the periplasm and the outer lipid membrane (Bretschger et al., 2007; Myers and Myers, 1992) (Figure 4A). In *L. monocytogenes*, a single protein, termed PplA, with two flavin molecules, enables the transport of electrons from the membrane to the exterior of the cell (Figure 4B).

The analyses of the distribution of the genes for this novel EET pathway revealed that EET activity likely occurred in an evolutionarily diverse subset of gram-positive bacteria, particularly in certain human gut bacteria, such as those of the genus *Lactobacillus*. Given that orthologs of the identified EET genes in *L. monocytogenes* are present in hundreds of species, this interesting aspect can be utilized for developing technologies based on EET capability of pathogens.

However, for other human electrogenic pathogens such as *Klebsiella pneumoniae*, *Enterococcus avium*, *Enterococcus faecalis*, *Capnocytophaga ochracea*, *A. actinomycetemcomitans*, *and P. gingivalis*, current production/mineral reduction mechanisms are unclear because they do not have any genes that encode conclusive or well-characterized EET pathways (Naradasu et al., 2018, 2020b; Pankratova et al., 2018; Zhang et al., 2020b). Recent studies have identified redox species at the cell surface and membrane in *S. mutans* (Naradasu et al., 2020a), *C. matruchotii* (Naradasu et al., 2020c), *A. actinomycetemcomitans*, and *P. gingivalis* (Naradasu et al., 2020b) by DAB staining and electrochemical analysis suggesting that EET might be involved in current production. The observed redox potential ranged from −125 mV to +250 mV, which is comparatively negative than those in the gut microbiome (Table 1). The more positive redox potential of gut pathogens may be more favorable for EET with distinct electron transfer pathways. However, genes responsible for these redox enzymes are still under investigation. Together, unlike environmental bacteria, the EET capability of pathogens has not well understood, and, therefore, not widely investigated yet. Nevertheless, the current production capability of these pathogens implies a lot of room to explore, which would give a paradigm shift in many aspects.

Although the involvement of cell-surface proteins is not yet clear, there are some reports on microbes that utilize soluble electron shuttle for current production as well (Keogh et al., 2018; Khan et al., 2012; Pankratova et al., 2018). The gram-positive lactic acid bacterium *Enterococcus* *faecalis* (*E. faecalis*), an opportunistic human pathogen, has shown electron transfer capability to electrodes directly and indirectly via mediators generated during fermentation. Comparative analysis of wild-type and mutant *E. faecalis* cells have showed that reduced dimethyl menaquinone in the respiratory chain in the bacterial cytoplasmic membrane is crucial for EET. Heme proteins were not involved, and cytochrome bd oxidase activity was found to attenuate EET. Another gram-negative opportunistic pathogen, *Pseudomonas* *aeruginosa* (*P. aeruginosa*), an aggressive biofilm builder that can live in various environments because of its ability to catabolize a large number of substances (Bassetti et al., 2018) and preferentially obtain its energy from aerobic respiration, can also use the anode as an electron acceptor to generate energy for active growth (Rabaey et al., 2005). It can use secreted phenazine derivatives as soluble mediators that largely enhance electrode-cell electron transfer, resulting in increased current generation. In addition, the diffusible phenazine derivatives enable the use of the electrode as an electron sink for cells in thick multilayer biofilms (Cornell et al., 2020). Another attractive phenomenon is that in a mixed culture, the secreted mediators can be used not only by *P. aeruginosa* itself but also by other microorganisms, which generally are not able to produce redox active mediators (Pham et al., 2008).

## The eco-physiological role of EET in electrogenic pathogens and biofilm formation

Understanding why pathogen-containing biofilms have EET capability is critical, as the fermentative metabolism of these biofilms does not require an electron acceptor substrate. Although respiration requires electron acceptors, the redox cycling of biological electron carriers, such as NADH, that drive intracellular oxidation and reduction of organic substrate can terminate fermentation. In fact, the coulombic value of microbial current production is far less than the total number of electrons generated from oxidation (i.e., the coulombic efficiency) (Doyle and Marsili, 2018). EET, therefore, may not only be involved in driving metabolism but also in regulation, sensing, or intercellular communication during fermentation by pathogens. Meanwhile, export of excess reductive substrate from the cell interior to facilitate fermentation may be one physiological role played by EET. In anaerobic environments with lower redox potentials (−500 to −200 mV), such as the human gut (Edwards et al., 1985), NAD^+^ regeneration is highly suppressed. In these environments, EET can increase the rate of NAD^+^ regeneration, and fermentative metabolism may be important to increase net energy gain, allowing pathogens to effectively compete with other respiratory bacteria. In fact, *S. mutans* showed almost no current production when pregrown at neutral pH with buffer, although pregrown condition at low pH caused significant *I*_*c*_ (Figure 2C). Given low pH is relevant to the biofilm condition of *S. mutans* (Guo et al., 2015), this observation may support our notion that EET is important for *S. mutants* to efficiently generate metabolic energy in the biofilm.

Reduced energy stress would also be critical for thicker biofilms composed of various microbes, in which EET may have a critical role. Long-distance microbial electron transport has been discovered in filamentous, multicellular cable bacteria from natural environments, such as marine sediments (Bjerg et al., 2016, 2018; Burdorf et al., 2016). These bacteria efficiently transport electrons from one end to another, spanning anaerobic and oxygenated environments. Drawing an analogy to this observation, EET from anaerobic to aerobic regions could help these pathogens survive in oxidant-limiting conditions by facilitating energy harvesting via inter-species electron transport across the biofilm.

**Figure 5:**
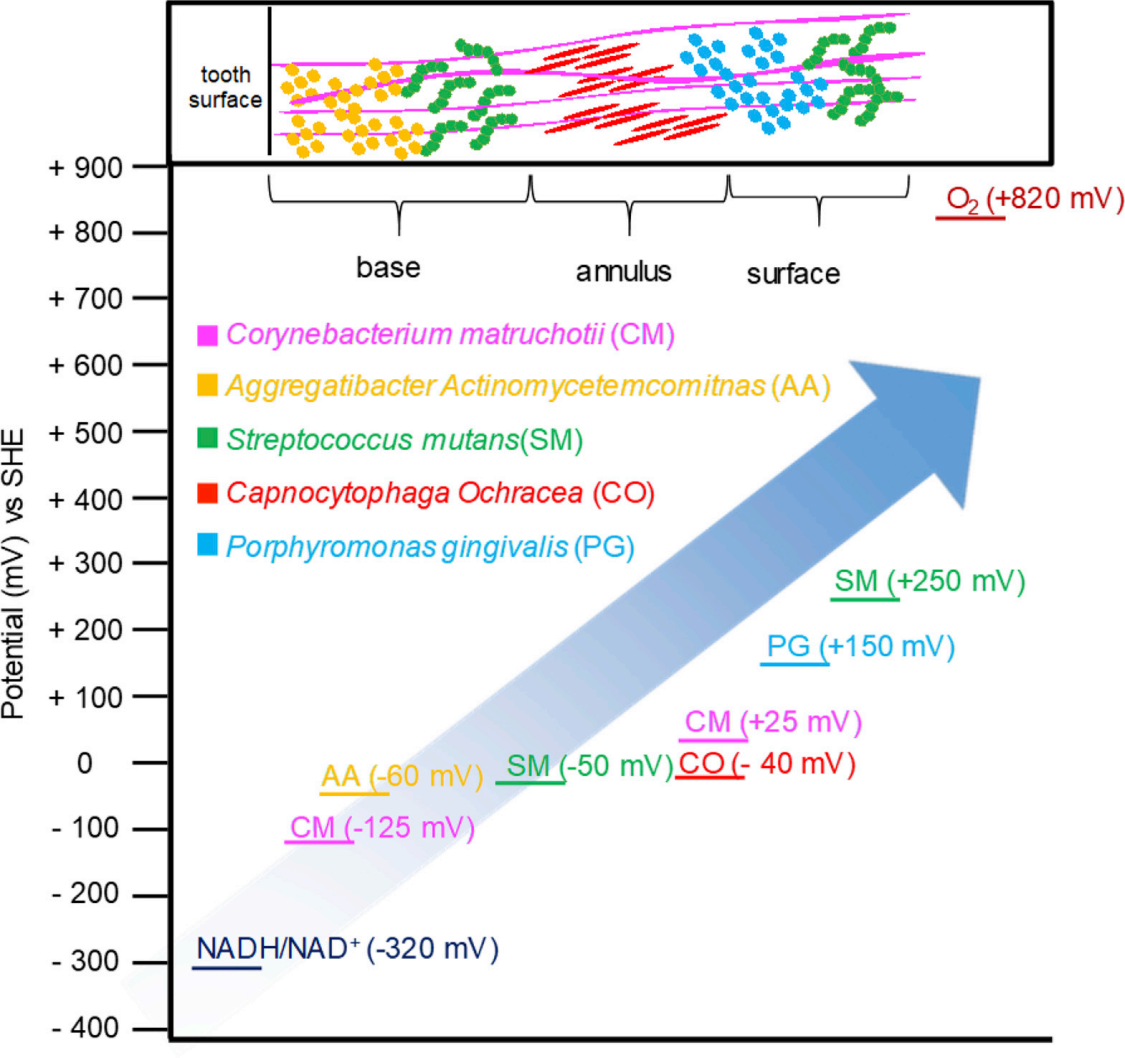
Arrangement of oral pathogens in the oral biofilm versus their cell-surface reagent redox potentials The architecture of the human oral biofilm representing the arrangement of oral pathogens from tooth base to surface (Welch et al., 2016). Energy diagram of identified current-producing oral pathogens based on observed cell-surface redox regents show that microbes near the tooth surface and the periphery have more negative and positive redox potential, respectively, suggesting that thermodynamic requirement for electron conduction across the polymicrobial biofilm via cell-surface redox species is fulfilled. CM: *Corynebacterium matruchotii*, AA: *Aggregatibacter actinomycetemcomitans,* SM: *Streptococcus mutans*, CO: *Capnocytophaga ochracea* PG: *Porphyromonas gingivalis.*

According to Welch et al., the oral microbiome is arranged in a manner that supports proliferation of anaerobes and aerobes in the oral plaque, which contains both oxygen and nutrient gradients (Welch et al., 2016). On the tooth surface, local oxygen gradients create anaerobic, anoxic, and aerobic interfaces, and biofilm span a range of more than 200 μm. Anaerobes exist in deep anaerobic spaces, facultative anaerobes in anoxic spaces, and aerotolerant bacteria near the surface—where plenty of oxygen is available (Welch et al., 2016). This oxygen gradient suggests the possibility of long-range EET in oral biofilms, where anaerobes deep in the biofilm can transfer electrons to the aerobic space for reduction by oxygen (as the final electron acceptor). The main members of the oral biofilm, *Streptococcus mutans*, *Capnocytophaga ochracea, Corynebacterium matruchotii*, *Aggregatibacter actinomycetemcomitans*, and *Porphyromonas gingivalis* (Naradasu et al., 2020a, 2020b, 2020c; Zhang et al., 2020b), can produce electric current with cell-surface redox species. Given cell-surface redox regents can mediate lateral electron transport across microbial aggregation (Okamoto et al., 2012; Chong et al., 2019), it is possible that these electrogenic pathogens can exchange electron in the polymicrobial biofilm. Therefore, the positions of each bacterial species in polymicrobial oral biofilm and their cell-surface redox potential are plotted in Figure 5. The energy levels show a cascade from the reductive inside to the oxidative outside, fulfilling a thermodynamic condition to have electron conduction through the biofilm to export the excess reductive energy from the anaerobic space. Electron conduction mechanism through thick biofilm may be a potential explanation for metatranscriptomic analysis that showed highly active anaerobic microbes for synthesizing mRNA even while biofilm conditions are unfavorable for anaerobic reductive metabolism (Benítez-Páez et al., 2014).

It is also possible that the pure culture biofilm of the electrogenic pathogen has electrical conductivity. In environmental EET-capable bacteria such as *Geobacter*, clonal biofilm has electric conductivity over distances on the centimeter scale (Malvankar et al., 2011; Yates et al., 2016). The pilin nanofilaments (microbial nanowires) produced by *Geobacter* have shown to assist conductance. Nanowire could be formed through the connection of membrane vesicles aligned (Deng et al., 2018; Subramanian et al., 2018). Electron conduction could occur even without such nano wire formation, and outer membrane cytochromes can mediate (Okamoto et al., 2012; Chong et al., 2019). Since many oral pathogens are electroactive and forms polymicrobial biofilm, such mechanism may take place in clonal pathogen biofilms as well.

## Other potential application of pathogen’s electrogenicity for EET-based biosensors

EET-based biosensors are studied for self-powered portable biosensing devices with potential for long-term and remote environmental monitoring. In environmental biosensing applications, analysis of the biological oxygen demand is one of the highly prevalent applications of EET-based biosensors in wastewater monitoring (Chang et al., 2004). In particular, the steady current output usually correlates with the concentration of the organic matter to certain level (Kumlanghan et al., 2008; Nakamura et al., 2007). Other than the biological oxygen demand, microbial current production was used to sense many other toxins such as heavy metals and organics, e.g., diazinon, polychlorinated biphenyls, p-nitrophenol, levofloxacin etc., (Kim et al., 2007; Wang et al., 2016; Zeng et al., 2017). These EET-based biosensors have helped in reducing the analysis time (minutes to hours) and efforts, compared with conventionally applied procedures.

To this end, EET-based biosensors may be highly suitable for monitoring the activity of microbiome in human and animal. Assuming pathogens are more electrically active in human gut environment, current production signals could reflect the abundance and activity of pathogens. Electrochemical biosensor with improved selectivity, sensitivity, and stability may make such opportunity. Given animal pathogens have shown to potentially carry out EET (Rago et al., 2021; Schwab et al., 2019; Wang et al., 2019b), the same electrochemical biosensor might be applicable to animals as well.

## Prospects and conclusions

To date, there are limited studies about EET capability of human pathogens (Table 1), but many studies have been published for EET capability of environmental bacteria, and applications close to our system such as environmental biosensors have been explored, demonstrating that basics of this field had been already established. Recent works extended the boundaries of this field to other niches such as human pathogens. Also, as highlighted, the orthologues of the identified EET genes in human pathogens are present in hundreds of species, thus EET activity likely occurred in an evolutionarily diverse subset of bacteria. Consequently, the present review will help in developing the research further aiming to assess and screen the effects of antimicrobials on biofilm activity by employing the current producing capability in pathogens.

Although human pathogens have been extensively characterized in disease studies, the capability of pathogens to produce electric currents remains largely uninvestigated. The discovery of electrogenic activity in pathogens, therefore, highlights the potential advantages of this classic technique, SA, for monitoring the metabolic activity of biofilms. The electrochemical reactor systems currently used for current production and drug testing involve relatively large volumes, making these systems infeasible for high-throughput screening of potential antimicrobials. Future studies should emphasize scaled-down systems, for example, using screen-printed electrodes or multi-well electrode systems (Molderez et al., 2020; Tahernia et al., 2020) that require minimum amounts of sample and antimicrobials. Such systems that exploit the electrogenic activity of pathogens could fast-track the discovery of antibiofilm drugs, as a large library of compounds could be rapidly screened. This concept could serve as a generally applicable technology for evaluating the efficacy of antimicrobials, as well as the selection of appropriate drugs or treatment regimens. Furthermore, recent findings related to bacterial electron production in mammalian gut suggest a new scope for interpreting the complex microbiology of gut bacteria and the effects of the gut microbiome on host physiology *in vivo.* It could also enable personalized drug sensitivity assessment by using microbiome from individuals on electrodes, which could aid the development of precision medicine. The reliability of SA-based drug discovery can be validated against one or more suitable conventional antibiotic testing methods where more detailed information is required.

A high-throughput system would also be effective for examining the mechanisms and physiological significance of EET. The relevance of EET to infections also warrants fundamental investigation because a better understanding of the mechanism underlying EET in pathogens could lead to the discovery of key target molecules for drug design and information about the design of materials for biomedical applications. Based on this, a strategy incorporating the monitoring of pathogenic activities *in vivo*, following therapeutic interventions, could be developed. Lastly, revealing the unexplored properties of well-studied bacteria that are critical for microbial metabolism could have a great impact on applied and fundamental scientific research.
